# Who Receives Home-Based Perinatal Palliative Care: Experience from Poland

**DOI:** 10.1155/2013/652321

**Published:** 2013-09-03

**Authors:** Aleksandra Korzeniewska-Eksterowicz, Łukasz Przysło, Bogna Kędzierska, Małgorzata Stolarska, Wojciech Młynarski

**Affiliations:** ^1^Pediatric Palliative Care Unit, Department of Pediatrics, Oncology, Hematology and Diabetology, Medical University of Lodz, 36/50 Sporna Street, 91-738 Lodz, Poland; ^2^Gajusz Foundation, Pediatric Palliative Care Center, Home Hospice for Children of Lodz Region, 87 Dąbrowskiego Street, 93-271 Lodz, Poland; ^3^Institute of Psychology, University of Lodz, 10/12 Smugowa Street, 91-433 Lodz, Poland; ^4^Department of Pediatrics, Oncology, Hematology and Diabetology, Medical University of Lodz, 36/50 Sporna Street, 91-738 Lodz, Poland

## Abstract

*Context*. The current literature suggests that perinatal palliative care (PPC) programs should be comprehensive, initiated early, and integrative. So far there have been very few publications on the subject of home-based PC of newborns and neonates. Most publications focus on hospital-based care, mainly in the neonatal intensive care units. *Objective*. To describe the neonates and infants who received home-based palliative care in Lodz Region between 2005 and 2011. *Methods*. A retrospective review of medical records. *Results*. 53 neonates and infants were admitted to a home hospice in Lodz Region between 2005 and 2011. In general, they are a growing group of patients referred to palliative care. Congenital diseases (41%) were the primary diagnoses; out of 53 patients 16 died, 20 were discharged home, and 17 stayed under hospice care until 2011. The most common cause of death (56%) was cardiac insufficiency. Neurological symptoms (72%) and dysphagia (58%) were the most common clinical problems. The majority of children (45%) had a feeding tube inserted and were oxygen dependent (45%); 39 families received psychological care and 31 social supports. *Conclusions*. For terminally ill neonates and infants, perinatal palliative care is an option which improves the quality of their lives and provides the family with an opportunity to say goodbye.

## 1. Introduction

Constant development and improvement of prenatal diagnosis present new opportunities in the field of perinatal palliative care (PPC) [1, 2]. The current literature suggests that PPC programs may be comprehensive, initiated early, and integrative [2]. PPC is understood as a constant care of a family during pregnancy and delivery and after the birth of its child and includes the care of newborns and infants [2–4]. 

In Poland, palliative care (PC) for children has been provided mainly by specialized pediatric palliative home-care teams. Such a home-based model of care is called “home hospice” [5]. Currently in Poland (38 million inhabitants), there are almost 40 pediatric home hospices, and in the years 2000–2010 the number of children treated in home hospices had increased more than fivefold [6]. Planned physician visits take place once a fortnight, and planned nurse visits twice a week. Patients and parents have a round-the-clock access to telephone consultation service, and intervention visits are a standard procedure in case of a patient's health deterioration. Visits of psychologists, social workers, physiotherapists, and hospital chaplains are planned according to individual needs of each family. PC is free of charge—all children have governmental health insurance. All medical equipment is lent to the families free of charge.

The Home Hospice for Children of Lodz Region (HHChLR) is a specialized pediatric palliative home care team, in this paper referred to as “home hospice.” HHChLR was founded in 2005. Since then it has had a total of over 180 children under its care and taken the fourth place in Poland in terms of the number of treated children. In the Lodz Region (over 2,5 million inhabitants with 471,000 children), there is only one other pediatric home hospice and HHChLR looks after 80% of children eligible for PC.

So far there have been very few publications on the subject of home-based PC of newborns and neonates [2, 4, 7]. Most publications focus on hospital-based care, mainly in neonatal intensive care units [8, 9].

The aim of this study is to describe the population of the neonates and infants who received home-based PC provided by HHChLR. 

## 2. Material and Methods

We conducted retrospective reviews of medical records to obtain data about all patients aged 0–12 months under the care of HHChLR between January 1, 2005, and December 31, 2011. 

The following variables were abstracted from the medical records: (1) demographic information (patient age and gender); (2) clinical information (principal diagnosis that triggered original referral to palliative care, main clinical symptoms, medications, and medical technology utilized); and (3) follow-up information (death or discharge from the hospice, time of death, location of death, duration of PC at home, and social and psychological support).

The principal diagnoses were divided into 4 groups according to International Statistical Classification of Diseases and Related Health Problems—10th Revision (ICD-10) [10]: (1) congenital malformations, deformations, and chromosomal abnormalities (CM); (2) certain conditions originating in the perinatal period (CCPP); (3) diseases of the nervous system (DNS); (4) metabolic diseases (MD). The location of death was categorized as home and hospital. The duration of care was calculated from the date of admission to the home hospice till the date of death or discharge from the home hospice or—for those patients who remained under home hospice care—until December 31, 2011. The study was approved by the Ethics Committee of the Medical University of Lodz (RNN/137/10/KE).

## 3. Statistical Analysis

Using the final database, descriptive statistics were used to describe patient characteristics. Data on the number of children (aged 0–19 years) living in the Lodz Region was downloaded from the databank of the Central Statistical Office of Poland [11]. The prevalence of children referred to the palliative care at home in Lodz Region was computed and converted to a denominator of 100,000. A linear trend was calculated using Pearson correlation coefficient for prevalence ratios of children aged 0–12 months and older than 12 months requiring PC in the respective years of the study. To determine differences between the groups in continuous variables, the Kruskal-Wallis test was used. Nominal variables were compared between the groups using a test for proportion with multicomparison Bonferroni correction. All statistical analyses were performed using STATISTICA for Windows release 8.0 software. Value of *P* < 0.05 was used as a definition of the statistical significance.

## 4. Results 

**Figure 1:**
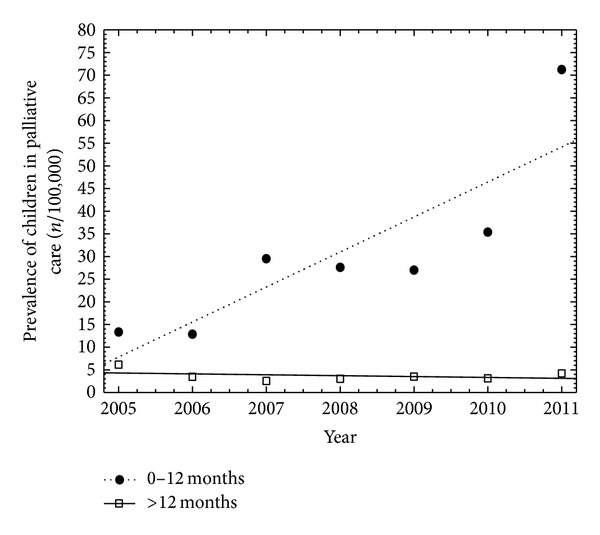
Prevalence of children referred to the palliative care at home in Lodz Region (years 2005–2011).

In the study period, 53 children aged 0–12 months (29% of all 184 patients) were admitted to HHChLR, and they are a growing group of patients referred to PC (Figure 1). At the time of admission to the home hospice the patients were aged between 24 and 357 days (median: 128; 25–75 percentile: 62–219,5); 51% of them were male. 

**Figure 2:**
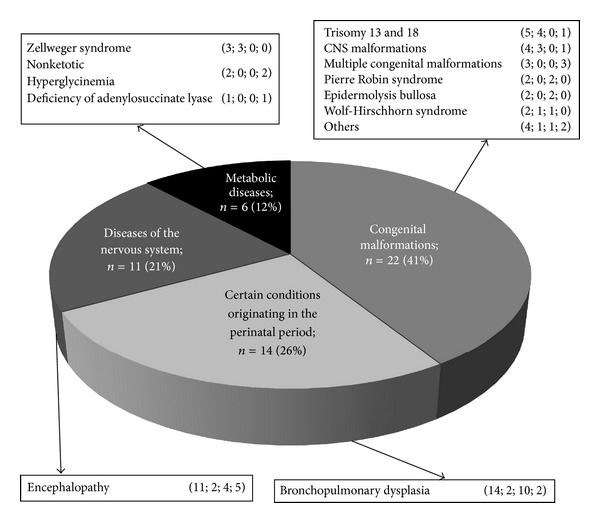
Diagnoses spanned a wide spectrum of patients' diseases. Numbers in parentheses indicate the total number of patients; the number of patients who died; the number of discharged patients; the number of patients who stayed under hospice care till December 31, 2011.

The most common principal diagnoses (41%) in the study group were CM; the longest duration of PC was observed in patients with DNS. Patients' characteristics are presented in Figure 2 and Table 1.

**Figure 3:**
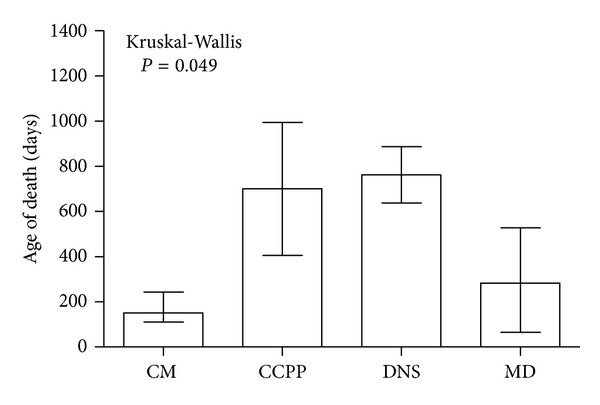
Age of death among the groups of study patients (expressed as median and 25–75 percentile). CM: congenital malformations, deformations, and chromosomal abnormalities. CCPP: certain conditions originating in the perinatal period. DNS: diseases of the nervous system. MD: metabolic diseases.

Out of the 53 patients admitted to the hospice, 16 died, 20 were discharged from home hospice and 17 were still under home hospice, care on December 31, 2011. The patients who died had been under home hospice care for between 10 and 751 days before their death (median: 136,5; 25–75 percentile: 78,6–253); ten patients died at home, 6 in a hospital. The highest mortality was observed among patients with MD but patients with CM died significantly earlier than those with other diagnoses (Figure 3). The most common causes of death were cardiac insufficiency (56%), respiratory insufficiency (16%), hemorrhage (16%), and cardiorespiratory insufficiency (12%). The location and causes of death did not differ between the groups of patients. 

The most common reason for being discharged from the home hospice was stabilization of health status. Discharged children suffered mainly from bronchopulmonary dysplasia and congenital diseases. The duration of PC at home among patients who were discharged varied from 10 to 727 days (median: 125,5; 25–75 percentile: 70,5–187,3).

**Figure 4:**
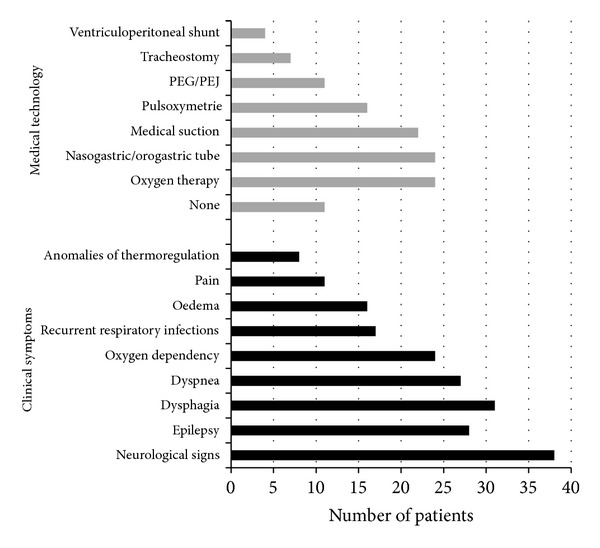
Clinical symptoms and medical technology used by study patients.

The most frequent clinical problems included neurological symptoms (72%) and dysphagia (58%)—Figure 4. The incidence of such symptoms was significantly lower among patients with CCPP than in the other groups (*P* < 0.001).

A high percentage of the infants (45%) had a feeding tube (naso- or orogastric) inserted and were permanently or periodically oxygen dependent (45%) (Figure 4).

**Figure 5:**
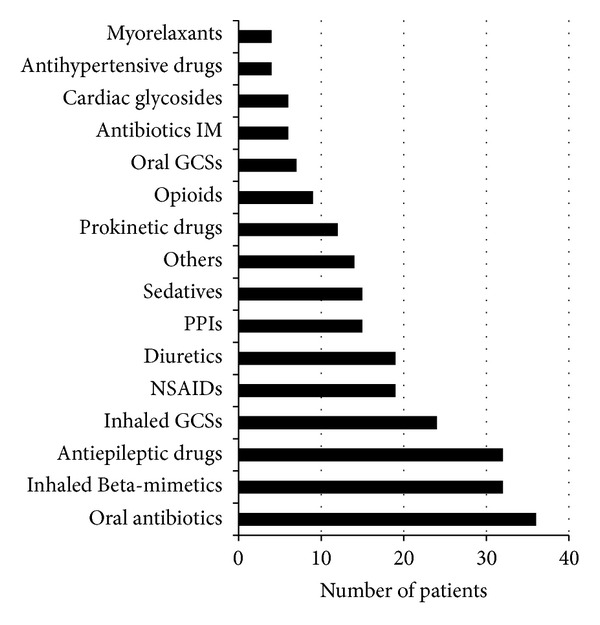
Groups of drugs used by study patients. GCSs: Glucocorticosteroids. NSAIDs: Nonsteroidal anti-inflammatory drugs. PPIs: Proton-pump inhibitors.

The subjects had extensive medication profiles: the mean number of medications per patient was 8.4 (SD 4.9), with a range from 3 to 26 medications—Figure 5. Palliative sedation, provided at home by subcutaneous access, was indicated in 7 patients, including 3 in the end stage of life and 4 during the hospice care. In all cases, midazolam was used as the primary medication; in 4 cases morphine and in 1 case buprenorphine were added to the treatment. Metoclopramide and hyoscine were used in 3 cases as adjuvants during sedation before death. There were no differences in medications prescribed between patients from different groups of principal diagnoses. Nevertheless, children with CCPP used no opioids, and a different profile of antiepileptic drugs (AEDs) was observed between the groups—Table 2.

According to the standard of care in HHChLR at least one visit of a psychologist and social worker took place in each family. Out of 53 families 39 received permanent psychological care and 31 social supports. Ten families of children who died participated in a support group for bereaved parents.

## 5. Discussion

Transition to PC is the most difficult decision for the parents of a child with life limiting illness. This decision is usually connected with many questions and fears, especially when care is planned as a home-based model. This model of PC should be promoted as the most suitable for both children and their families, as well as cheaper than the inpatient model of care. This opinion corresponds with international standards which recommend home-based medical care with strong emphasis on psychological and social support [12, 13]. The largest so far retrospective studies conducted to date, in the United States [14] and in Great Britain [15], describe a significant change over time in the place where most children with a life limiting illness die. Earlier research conducted by HHChLR indicated a strong need for that type of care in Lodz Region, especially in the field of PPC [16]. We showed that in the years 2000–2004 almost 90% of children with chromosomal aberrations died in hospitals [16]. The current presented analysis shows that every third patient referred to HHChLR between 2005 and 2011 was under the age of 12 months. Additionally, we observed that the youngest patients were a growing group of those referred to PC at home. These results are consistent with nationwide observations, which may indicate a better cooperation between hospices and neonatal and prenatal diagnostic centers [6].

The ideal model of PC for children prenatally diagnosed with lethal defects includes hospice care during pregnancy. Polish law allows pregnant women whose fetus has been diagnosed with a lethal defect to perform abortion up to 22–24 weeks of the pregnancy gestation. However, abortion is a challenging decision and hard to discuss in Poland, a country where 90% of the population are declared catholics. From the experience of HHChLR, it seems that those children with lethal defects who survive after birth are very often in need of invasive treatment and frequently die in the hospital. Epidemiological data from the Lodz Region in 2010 showed that 101 children died before 12 months of age, including 80 newborns, and the present study showed that the youngest patient referred to home PC was 24 days old. Home based PPC is therefore an alternative for those families who decide not to terminate the pregnancy, and it protects the child from suffering and risky medical interventions [17, 18]. Multidisciplinary care allows the family to say goodbye to their terminally ill baby in a peaceful environment. 

The above described model of care in case of an unsuccessful prenatal diagnosis is currently being discussed in the Lodz Region. The HHChLR team is promoting this model through specialised training offered to pediatricians and nurses. That work has been inspired by the current experience of HHChLR—all the families who took part in this research received postnatal PC notwithstanding the prenatal diagnosis. None of the families had been informed during the pregnancy about the possibility of PPC.

The analysis of patients referred to the HHChLR before their first birthday in terms of the primary diagnosis confirms the observations of other authors regarding the criteria for PC [3, 4, 7, 19, 20]. Some uncertainties in those matters can derive from the fact that there are cases of patients discharged from the home hospice due to the improvement of their clinical condition. Discharged children had been diagnosed mainly with bronchopulmonary dysplasia or had been waiting for surgical correction of congenital malformations. Very often home hospice care is the only alternative to the hospital care for those children, hence the referral. Such a procedure reflects some recommendations [1, 2]. The experience of HHChLR indicates that professional, constant home care improves the patients' general well-being and stabilizes their condition. The discharge from the home hospice is possible after the parents have been trained, family doctors informed about the child's condition, and the family provided with specialized medical equipment indispensable to the child.

The present data show that the longest PC was provided for patients with neurological diseases and the primary clinical symptoms were neurological signs and dysphagia. Given the profile of the patients, these results should not be surprising and are consistent with observations of other researchers [3, 4, 19].

It is known that the occurrence of neurological symptoms is not limited only to the diseases of nervous system, and unfortunately ICD-10 does not give a full scope of those diseases qualifying for PC. Most metabolic diseases are in fact neurometabolic; many patients with chromosomal defects and congenital malformations display pathology of their neurological systems [21, 22]. Thus, the first year of life is the period with the highest incidence of epilepsy in children and adolescents. It is worth noting that ICD-10 classifies epilepsy as a separate disease where in fact it is a range of somatic, psychic, and vegetative symptoms resulting from many metabolic and morphological changes in the brain. In the present study different profiles of AEDs were observed between the groups. Among the patients with epilepsy, those in palliative care are more often patients with drug-refractory epilepsy. The causes of intractable epilepsy are neurometabolic disease, disorders of brain development, and specific epileptic encephalopathies [23]. Additionally, the situation of infants is disadvantageous especially because of the small number of registered AEDs. Thus, the control of drug-resistant epilepsy in this population is often inadequate, and requires a rational and appropriate polytherapy, often with the use of old generation AEDs [24, 25]. The care of patients with a chronic and often progressive neurological disease is complex and requires a multilevel approach. To provide the patient and his/her family with adequate care and support, the most appropriate form of care seems to be home pediatric PC.

Dysphagia caused by the motility abnormalities of the upper part of digestive system in children with severe CNS damage can occur anytime during a child's life. During the infant stage, those patients with chromosomal abnormalities, congenial CNS malformations, and severe HIE present abnormalities of sucking and swallowing reflexes [26, 27]. Total enteral feeding with the appropriate management of other symptoms leads to a fast improvement of the children's condition, which confirms the diagnosis and builds the parents' trust. The present study showed that 45% of patients needed nutritional support before the age of one. The type of support is conditioned above all by the diagnosis and prognosis as well as the length of life [27]. In addition, the type of medical technology used reflects the profile of patients referred to PPC. Almost half of the patients presented oxygen dependency, and one in ten patients were provided with tracheostomy or ventriculo-peritoneal shunt. The youngest patients of HHChLR did not have central venous catheters and noninvasive ventilation. 

Some findings connected with the medications profile warrant emphasis and discussion. A particular issue is the use of palliative sedation at home. Continuous palliative sedation (CPS) is usually implemented at the end of life, but the definition of CPS does not preclude its application earlier if persistent symptoms intolerable for the patient are observed. In the present study, both situations were an indication for this procedure. Although there is no shortage of data on the reasons for and implementation of CPS in adult patients, the number of studies in the pediatric population is small [28–30]. Our observations support the view that CPS can be utilized at any age, including newborns and infants, taking into account distinct pharmacokinetic and pharmacodynamic properties of sedative drugs. Seven years of HHChLR experience (CPS provided in over 40 patients—unpublished data) indicate that palliative sedation can be safely used in the patient's home, on condition that the guardians are instructed how to operate the equipment, supply the drugs and monitor sedation.

PC includes the bereavement phase of the family life as well [1, 2], and our observations confirm the need for relevant support. 

The limitations of the present study include the lack of comparison with other models of PC since home-based care is dominant in Poland. There is also a lack of similar research in Poland. Another limitation is the small number of participants. However, the study describes 7 years of experience with the home-based model of PPC provided by HHChLR, which looks after 80% of families referred to palliative care in the Lodz Region; therefore, our results can be considered as fairly reliable and representative for the entire region.

## 6. Conclusions

In cases where the prognosis remains uncertain, PPC is an option which improves the quality of patients' life and provides the family with an opportunity to say goodbye to them in a peaceful way. The results of the present study are in accordance with the suggestions in the current literature that PPC programs should be comprehensive, initiated early and integrative.

## Figures and Tables

**Table 1:** Diagnoses spanned a wide spectrum of patients' diseases and patients' characteristics.

Patients' groups according to diagnoses	*N* (%)	Age of admission in days median (25–75 percentile)	Deaths		Discharges		Patients remaining under hospice care on December 31, 2011.	
			*N *	Age of death in days median (25–75 percentile)	*N *	Duration of care in days median (25–75 percentile)	*N *	Duration of care in days median (25–75 percentile)
Total	53 (100)	128 (62–219,5)	16	248 (138–528)	20	125,5 (70,5–187,3)	17	264 (161–833,5)
Congenital malformations, deformations, and chromosomal abnormalities	22 (41)	82 (38,5–191,8)	9	150,5 (110,3–242,8)	6	218 (104,3–404,5)	7	264 (170–549)
Certain conditions originating in the perinatal period	14 (26)	139 (119,3–204,5)	2	700,0405,0	10	111 (58,75–144,3)	2	52 (52–52)
Diseases of the nervous system	11 (21)	91 (55–314)	2	762 (637–887)	4	157,5 (41–186,3)	5	814 (226,5–1346)
Metabolic diseases	6 (12)	187 (139,8–249)	3	283 (65–528)	—	—	3	344 (23–1921)

**Table 2:** Profile of antiepileptic drugs used by study patients.

Antiepileptic drugs	Groups of the principal diagnoses									
	Congenital malformations, deformations, and chromosomal abnormalities (*n* = 22)		Certain conditions originating in the perinatal period (*n* = 14)		Diseases of the nervous system (*n* = 11)		Metabolic diseases (*n* = 6)		Total (*n* = 53)	
	*n *	%	*n *	%	*n *	%	*n *	%	*n *	%
Phenobarbital	12	54	3	21	9	82	5	83	25	47
Diazepam	11	50	1	7	10	91	4	66	28	53
Vigabatrin	—	—	1	7	6	54	3	50	10	19
Topiramate	1	4	1	7	3	27	3	50	8	15
Lamotrigine	—	—	—	—	1	9	1	16	2	4
Valproic acid	2	8	1	7	5	45	4	66	12	23
Clonazepam	2	8	—	—	—	—	2	32	4	8
Zonisamide	—	—	—	—	—	—	1	16	1	2
Clomethiazole	—	—	—	—	—	—	1	16	1	2
Nitrazepam	—	—	—	—	1	9	—	—	1	2
